# Clinical outcomes of agalsidase Beta (fabrazyme) in Chinese fabry disease patients with proteinuria: a case series

**DOI:** 10.3389/fped.2026.1839303

**Published:** 2026-06-17

**Authors:** Zelei He, Yuanyuan Wu, Hongmei Yang, Yijuan Li, Lingling Xu

**Affiliations:** Department of Pediatrics, The First Affiliated Hospital of Sun Yat-Sen University, Guangzhou, Guangdong, China

**Keywords:** agalsidase beta, case series, Chinese population, enzyme replacement therapy, fabry disease, renal transplantation

## Abstract

**Background:**

Fabry disease (FD) is a rare X-linked lysosomal storage disorder caused by pathogenic *GLA* gene mutations, leading to deficient *α*-galactosidase A (*α*-GalA) activity and systemic accumulation of globotriaosylceramide (Gb3) and its derivatives. It is characterized by progressive multi-organ damage, especially nephropathy and cardiomyopathy. Agalsidase beta (Fabrazyme; Sanofi, Paris) for enzyme replacement therapy (ERT) has been approved in China. We report the clinical outcomes of two Chinese adult male FD patients with proteinuria treated with agalsidase beta.

**Cases presentation:**

Two Chinese male patients with classic FD phenotypes and proteinuria were treated with agalsidase beta (1 mg/kg every two weeks). Case 1 (last followed up at age 34,*GLA* c.493G > T mutation) developed proteinuria at age 23, presented with severe renal impairment, and underwent kidney transplantation at approximately age 32 after receiving ERT for 21 months prior to transplantation. Post-transplant, his renal function stabilized (creatinine <115 μmol/L) with resolved proteinuria.Renal recovery was primarily attributed to transplantation, while ERT contributed to systemic control. His plasma Lyso-Gb3 decreased by 70.6% (93.33 to 27.38 ng/mL). Case 2 (last followed up at age 40, GLA c.1201T > C mutation) developed proteinuria at age 30, had stage 2 chronic kidney disease and left ventricular hypertrophy. Following genetic confirmation at age 36, he initiated ERT at age 37, with initial irregular dosing due to social factors and economic pressure, then transitioned to standardized biweekly dosing at age 38. After approximately 4 years of ERT, his Lyso-Gb3 decreased by 68.5% (79.17 to 24.93 ng/mL). His proteinuria improved (2195.52 to 1834.34 mg/24 h) with unchanged RAAS blocker doses. Both patients also experienced regression of some symptoms, including neuropathic pain and gastrointestinal discomfort, and tolerated the therapy well with no infusion-related adverse events.

**Conclusions:**

In these two cases, agalsidase beta was well-tolerated and associated with stabilization of renal and cardiac function. The management of proteinuria required concomitant RAAS inhibition. Family cascade screening remains critical for early diagnosis.

## Introduction

Fabry disease (FD, OMIM #301500) is an X-linked lysosomal storage disorder caused by pathogenic variants in the *GLA* gene, leading to deficient *α*-galactosidase A (*α*-GalA) activity and subsequent systemic accumulation of globotriaosylceramide (Gb3) and its deacylated derivative, lyso-globotriaosylsphingosine (Lyso-Gb3) (1–3). Reported birth prevalence varies widely depending on the population studied, ranging from 1 in 40,000 for the classic phenotype in males to as high as 1 in 3,000 when including later-onset forms and females identified through newborn screening (2, 3). Progressive multi-organ damage—particularly nephropathy, cardiomyopathy, and neuropathy—defines its clinical trajectory (2, 3). Notably, Lyso-Gb3, a biomarker of disease severity, directly induces podocyte injury and renal fibrosis, contributing to end-stage renal disease (ESRD), which accounts for the leading cause of FD-related mortality (4, 5).FD remains a significant therapeutic challenge, although current treatments have markedly improved outcomes. Enzyme replacement therapy (ERT), the primary treatment for FD, includes agalsidase-α (derived from human fibroblasts) and agalsidase beta (Fabrazyme, produced in Chinese hamster ovary cells) (6). The 2017 KDIGO conference highlighted ERT's role in alleviating cardiovascular and renal complications (7). However, long-term data specific to Chinese patients remain limited (8). Studies demonstrate that treatment with agalsidase beta at the approved dose of 1 mg/kg body weight every other week significantly reduces plasma globotriaosylceramide (Gb3) levels (6), decreases Gb3 deposition in critical organs (e.g., heart and kidneys), mitigates organ-related symptoms, lowers mortality risk, and improves patients’ quality of life. Additionally, agalsidase beta has been shown to alleviate gastrointestinal symptoms and neuropathic pain (9).Agalsidase beta (Fabrazyme) was approved in China in 2019. While long-term data on the safety and efficacy of agalsidase beta exist globally, published clinical experience in Chinese patients, particularly those with established renal involvement, remains limited. This report aims to contribute a reference point for FD treatment in China and throughout Asia.

## Methods

All clinical, laboratory, and imaging assessments were performed using standardized institutional protocols to ensure consistency and reproducibility. Cardiac assessment was performed using echocardiography according to the American Society of Echocardiography guidelines and renal evaluation used standardized eGFR, 24 h urinary protein. Lyso-Gb3 and *α*-GalA activity were measured using consistent LC-MS/MS and fluorometric assays, with intra-assay CV <5% and inter-assay CV <8%, ensuring longitudinal consistency of biomarker measurements.

### Case report

#### Case 1

**Figure 1:**
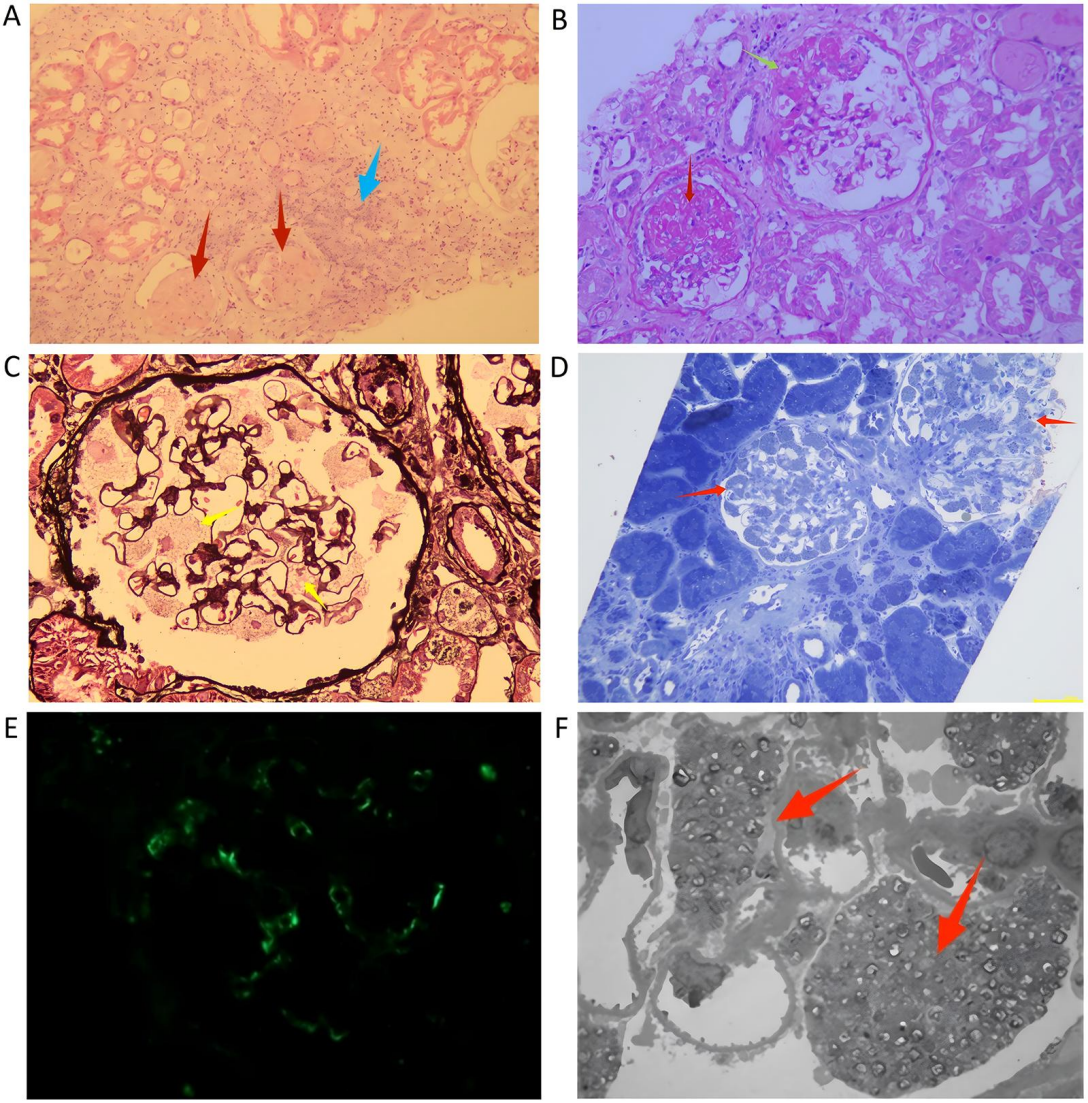
Light Microscopy, immunofluorescence, and electron microscopy findings of renal biopsy in case 1 (diagnosed at age 28). **(A)** Hematoxylin-eosin (HE) staining (×100). **(B)** Periodic acid-Schiff (PAS) staining (×200). **(C)** Periodic acid-silver methenamine (PASM) staining (×400). **(D)** Toluidine blue staining (×400). **(E)** Immunofluorescence staining for IgG, IgA, IgM, C3, C4, and C1q (all negative). **(F)** Transmission electron microscopy [×(insert magnification)]. Arrow legends: **(A)** Red arrows, global glomerulosclerosis; blue arrow, interstitial inflammatory cell infiltration. **(B)** Red arrow, global glomerulosclerosis; green arrow, segmental glomerulosclerosis. **(C)** Yellow arrows, podocyte swelling with vacuolated cytoplasm. **(D)** Red arrows, interstitial lipid deposits. **(E)** No immune complex or complement deposition. **(F)** Red arrows, numerous zebra bodies (myeloid bodies) within podocyte cytoplasm.

A Chinese male (last followed up at age 34), reporting a history of hypohidrosis since early childhood and a progression of distal extremity neuropathic pain for over 20 years. Since early childhood, he exhibited hypohidrosis and elevated skin temperature in the extremities. By age 9, he developed burning and tingling paresthesias in the distal limbs with variable intensity, followed by irregular bowel movements at age 10. Extremity pain gradually subsided by age 20. At 22, he experienced intermittent blurred vision, and proteinuria was detected at age 23. By 27, he reported bilateral tinnitus and postprandial left lower quadrant pain, followed by skin pruritus and brown plaques at age 28. Renal biopsy at age 28 confirmed Fabry nephropathy (Figure 1). By 30, he presented with frothy urine suggestive of proteinuria, loose stools, and transient precordial tingling episodes resolving spontaneously.

Physical examination at diagnosis (age 28–30) revealed a blood pressure of 149/93 mmHg, multiple variably sized brown round plaques with scattered scaling and rough surfaces on the trunk/limbs, and 2-mm non-blanching erythematous papules over the lumbar/abdominal regions. A pinpoint rash was observed on the right upper limb (little finger and metacarpophalangeal joints). Sensory disturbances with mildly heightened tactile sensitivity involved bilateral wrists, 10 cm proximal to the ankles, and the proximal interphalangeal joints of hands/first-second toes. Skin temperature asymmetry was noted (left foot cooler, right warmer).

Laboratory data at age 29 showed severe renal dysfunction (creatinine 430 μmol/L, urea 14.32 mmol/L, estimated glomerular filtration rate (eGFR, by CKD-EPI equation) of 15.0 mL/min/1.73m^2^, proteinuria 3.35 g/24 h), supported by renal ultrasound showing chronic kidney disease features. By 31, cardiac ultrasound revealed mild interventricular septal thickening (IVSd 12 mm), while audiometry detected right-sided mild sensorineural hearing loss. Fundoscopy indicated retinal vascular abnormalities.

Genetic testing detected a GLA missense variant (c.493G > T, p.Asp165Tyr). Biochemical assays confirmed severe *α*-GalA deficiency (0.37 vs. 2.4–17.65 µmol/L/h) and elevated lyso-Gb3 (93.33 ng/mL, normal <1.11 via MSMS), establishing Fabry disease. Familial screening revealed positive diagnoses in the patient's mother and daughter.

Post-diagnosis, carbamazepine was initiated for pain management. At age 30, biweekly agalsidase beta (1 mg/kg) was started. Due to progressive renal failure, peritoneal dialysis was initiated at age 31. After 21 months of ERT (at approximately age 32), right kidney transplantation was performed for CKD stage 5 and hypertension. ERT continued post-transplantation.

**Figure 2:**
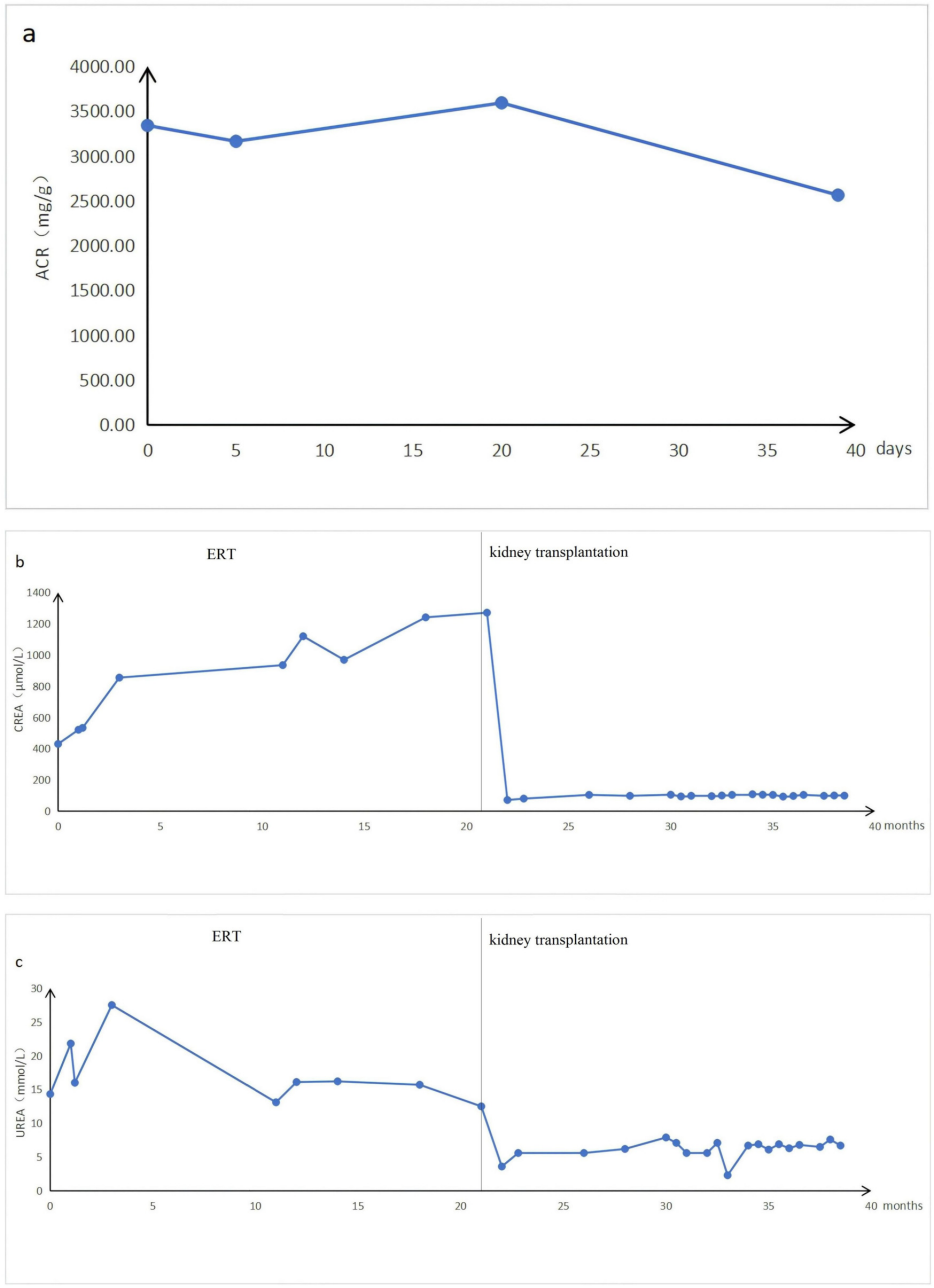
Renal function parameters in case 1 (last followed up at age 34). **(a)** Short-term dynamics of urinary microalbumin-to-creatinine ratio (UACR, normal <30 mg/g) during the first 40 days of ERT initiation. **(b,c)** Longitudinal changes in serum creatinine and blood urea nitrogen (BUN) levels. The vertical dashed line at Month 21 indicates the time of kidney transplantation, which was performed after 21 months of ERT. ERT was initiated at Month 0 (age 30).

Peritoneal dialysis led to creatinine rebound and persistent urea fluctuations (Figures 2b, c). Post-transplant, creatinine (<115 μmol/L) and urea (<8.6 mmol/L) normalized (Figures 2b, c), with resolved proteinuria and stable BP (115/65 mmHg). The normalization of renal function was mainly attributed to kidney transplantation; continuous ERT contributed to reducing systemic Lyso-Gb3 and preventing Gb3 redeposition in the graft, rather than reversing native renal failure.

Concomitant medications included carbamazepine for pain and post-transplantation immunosuppression with tacrolimus and mycophenolate mofetil and antihypertensives.

Following 4.2 years of ERT, Lyso-Gb3 decreased to 27.38 ng/mL (70.6% reduction). Pain improved markedly (VAS score from 7 to 1). Hypohidrosis, gastrointestinal symptoms, heat intolerance, visual disturbances, and right-sided tinnitus all improved. Angiokeratomas remained stable. Transplanted kidney function, blood pressure (115/65 mmHg), and cardiac parameters (IVSd reduced from 12 mm to 11 mm) were within normal ranges.

#### Case 2

A Chinese male (last followed up at age 40, initially presenting at age 36) reported a 30-year history of nonpruritic left inner thigh rash (non-blanching pinpoint erythema), proteinuria, and a 1-year chest tightness. His clinical history included a red pinpoint rash on the left inner thigh without itching or pain and discoloration when pressed, childhood-onset hypohidrosis (age 8) and chronic diarrhea (loose stools from age 15). Proteinuria was first detected at age 30. At age 35, cardiac ultrasound revealed left ventricular hypertrophy (LVH), which raised clinical suspicion.

Genetic confirmation of Fabry disease was obtained at age 36.RAAS blockade (valsartan 80 mg qd and empagliflozin 10 mg qd) was initiated immediately after diagnosis at age 36. ERT was started one year later at age 37. Due to social factors and economic pressure, the initial dosing was irregular (1 mg/kg every 3–4 weeks), which temporarily attenuated biomarker reduction and treatment efficacy, highlighting the importance of treatment adherence for optimal outcomes. At age 38, the patient transitioned to standardized biweekly dosing (1 mg/kg, approximately 70 mg based on body weight), which was well-tolerated without adverse events.

**Figure 3:**
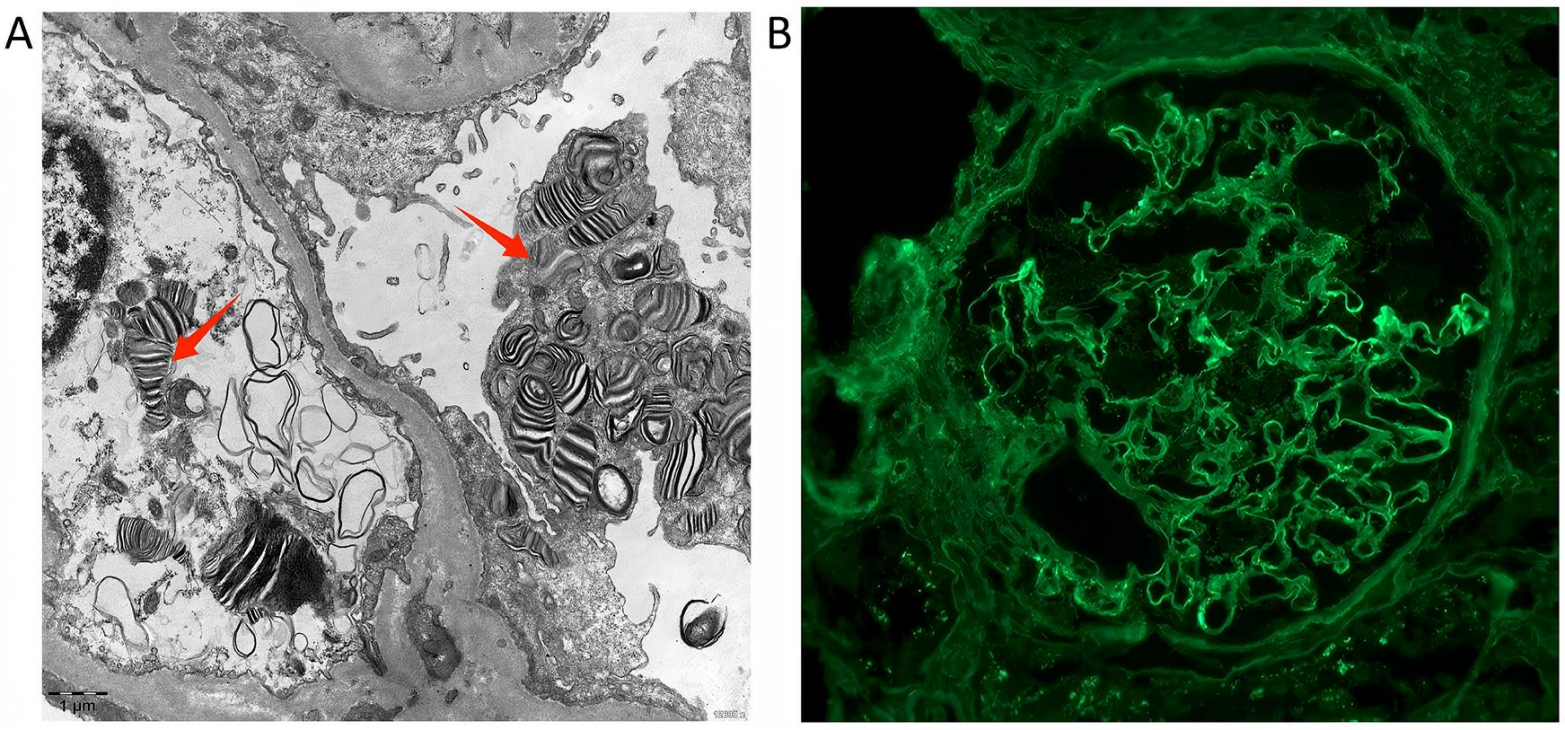
Electron microscopy and immunofluorescence findings of renal biopsy in case 2 (diagnosed at age 36). **(A)** Transmission electron microscopy showing numerous zebra bodies (myeloid bodies) within podocytes [×(insert magnification)]. **(B)** Immunofluorescence staining for immunoglobulins (IgG, IgA, IgM) and complement components (C3, C4, C1q) showing no immune complex deposition. Arrow legends: **(A)** Red arrows, zebra bodies (myeloid bodies) in podocyte cytoplasm.

Physical examination: At age 36, renal biopsy suggested Fabry nephropathy (Figure 3), with normal renal function but elevated proteinuria (2195.52 mg/24 h). Echocardiography at age 38 demonstrated mild septal/posterior wall thickening (IVSd 12 mm, LVPW 12 mm) with preserved systolic/diastolic function. Cerebral MRA at age 39 revealed hypoplasia of the right posterior cerebral artery P3-04 segment, consistent with a congenital vascular anomaly.

Genetic testing detected a pathogenic GLA missense variant (c.1201T > C, p.Ser401Pro). This variant was predicted to be probably damaging by PolyPhen-2 (HumDiv score 0.974). Biochemical assays confirmed severe *α*-GalA deficiency (0.37µmol/L/h, normal: 2.4–17.65) and elevated lyso-Gb3 (79.17 ng/mL, normal <1.11 via MSMS), establishing Fabry disease. Familial screening revealed positive diagnoses in his brother and daughter.

Following approximately 3.6 years of ERT, Lyso-Gb3 decreased to 24.93 ng/mL (68.5% reduction). Clinical improvements included partial regression of angiokeratoma, reduced proteinuria (from 2195.52 to 1834.34 mg/24 h), and improved hypohidrosis and diarrhea. Neuropathic pain improved (VAS score from 5 to 1), and hypohidrosis/gastrointestinal symptoms improved from “severe” to “mild” on a 5-point Likert scale. Cardiac remodeling showed slow progression (IVSd/LVPW from 12 mm to 13 mm).

## Discussion

Current literature on the efficacy and safety of agalsidase beta in FD patients with proteinuria as the predominant symptom remains sparse globally (Table 1). A PubMed search (keywords: Fabry disease, ERT, proteinuria; filtered by case reports, 2000-2023) yielded 13 publications, with 5 meeting inclusion criteria after manual screening. Fabry disease exemplifies the challenges of genotype-phenotype discordance, where identical *GLA* mutations can manifest with divergent clinical trajectories.This variability may be explained by epigenetic modifiers, environmental factors, residual *α*-GalA activity, and cellular stress responses such as the unfolded protein response (UPR) induced by misfolded mutant proteins, which can independently contribute to cellular dysfunction (10–14). This variability, emphasized in KDIGO guidelines, contributes to diagnostic delays averaging 10–15 years—a critical barrier to early intervention.

**Table 1:** Adult fabry disease cases with proteinuria receiving intravenous agalsidase beta.

Reference	Stanisława Bazan-Socha (33)		S. Taneda (34)	Bun Sheng (35)		Andy Sing Ong Tang (36)	Shinji Kume (37)	Case 1 (This study)	Case 2 (This study)
Years	2007		2013	2020		2021	2022	2025	2025
Age/Sex	48y/M	46y/M	46y/M	44y/M	52y/F	46y/M	48y/F	34y/M at last follow-up	40y/M at last follow-up
Age of diagnosis	38y	36y	43y	40y	48y	39y	36y	28y	36y
Mutant gene	1235del115/insA		NR	p.N215S c.644A > G [p.Asn215Ser]		c.610 T > C (p.Trp204Arg)	c.288G > A (p.Met96Ile)	c.493G > T(p.Asp165Tyr)	c.1201T > C(p.Ser401Pro)
*α*-GalA (*μ*mol/L/h)	0.12↓	0.20↓	↓	0.27↓	1.07↓	0↓	0.2↓	0.37↓	0.37↓
Lyso-Gb3 (ng/mL)	NR	NR	↑	1.17↑	Normal	22.0↑	NR	93.33↑	79.17↑
Initial symptom	Glomerulonep-hritis	Sudden hearing impairment	Renal insufficiency	Proteinuria (12.9 g/d)	Proteinuria (1.25 g/d)	Proteinuria (1 g/d)	Proteinuria (2.25 g/gcr)	Proteinuria; Acroparesthesias	Proteinuria; Skin rash
Main symptoms	Proteinuria (4.7 g/d); Angiokeratoma; Eye changes; Paresthesia	Mild proteinuria (0.78 g/d); Angiokeratoma; Lymphoedema of the lower limbs; Pre-excitation syndrome; Myocardial hypertrophy	Renal dysfunction before and after renal transplantation, including proteinuria, presence of mulberry cells and increase of the serum creatinine	Proteinuria; Progressive lower limb edema; Hypertrophic cardiomyopathy	Proteinuria	Proteinuria and edema; Cornea-verticillata; Angiokeratoma	Proteinuria (2.25 g/gcr); hyperuricemia	Proteinuria; Angiokeratoma; Hypohidrosis; Tinnitus	Proteinuria; Angiokeratoma; Diarrhea; Left Ventricular Hypertrophy
Renal transplantation	No	No	Yes, before ERT	Yes, after ERT	No	No	No	Yes(during ERT)	No
First time of ERT	43y	41y	About 44y	40y	48y	44y	36y	30y	37y
Dosage and Duration	Agalsidase beta 1.0 mg/kg q2w For four and a half years		Agalsidase beta 1.0 mg/kg q2w For 16 times	Agalsidase beta 1.0 mg/kg q2w For four years		Agalsidase beta 1.0 mg/kg q2w For two years	Agalsidase alfa 0.2 mg/kg q2w for 6 years→Agalsidase beta 1.0 mg/kg q2w for 6 years→+losartan (25 mg/day) for 10 years→+febuxostat for 1 years	Agalsidase beta 1.0 mg/kg q2w For 4.2 years	Agalsidase beta 1.0 mg/kg q2w (irregular 1.5y -> standard 2.1y) Total 3.6 years
Adverse Events	Shivers and dyspnea during infusion	Not reported	Not reported	Not reported	Not reported	Not reported	Not reported	No	No
Treatment outcomes	Improvement observed in all aspects, particularly in the stabilization of kidney function, cardiac hypertrophy and CNS abnormalities.		Improved. 1.Mulberry cells disappeared within 1 month of the initiation of ERT. 2.A slight lowering of the serum creatinine level. 3.low-level proteinuria was still detected.	Improved in heart and Lyso-Gb3, but not proteinuria before renal transplantation.	Improved in proteinuria (0.42 g/d).	Proteinuria and renal function remained stable.	1.Agalsidase alfa: no significant change in proteinuria; cardiac hypertrophy progressed. 2.Agalsidase beta: proteinuria was refractory; mild elevation in systolic blood pressure. 3.+losartan: systolic blood pressure improved, proteinuria remained unchanged. 4.+febuxostat: eGFR showed slight recovery.	Renal function stable post-transplant; Lyso-Gb3 decreased 70.6%; Pain improved.	Proteinuria improved; Lyso-Gb3 decreased 68.5%; GI symptoms improved.

**Figure 4:**
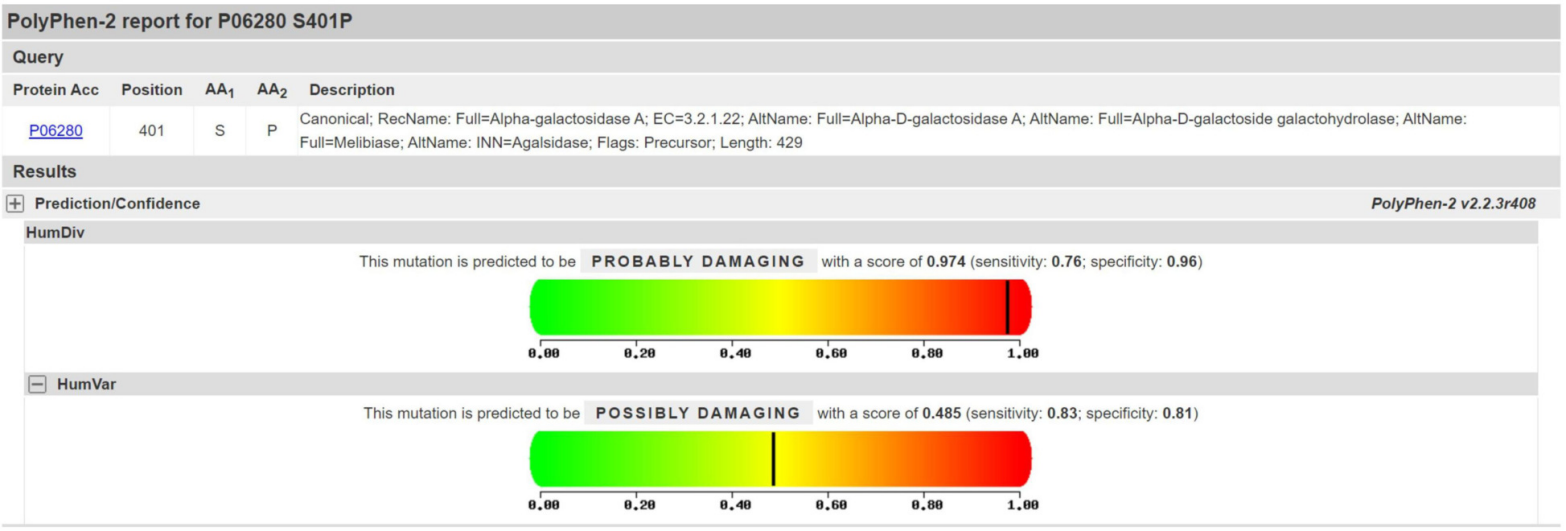
In silico pathogenicity prediction for the *de novo* GLA variant (NM_000169.2:c.1201T > C; p.Ser401Pro) identified in Case 2. PolyPhen-2 analysis using **(A)** HumDiv model (score: 0.974, “probably damaging”) and **(B)** HumVar model (score: 0.485, “possibly damaging”). The horizontal dashed lines indicate the classification thresholds.

The *GLA* c.493G > T variant identified in Case 1 is registered in the Fabry Database (http://fabry-database.org/mutants/), however, the clinical data remains scarce. Although this mutation has been reported, the associated phenotypes varied (Table 2). Case 2 involved a *de novo* mutation, which was predicted to be probably damaging by PolyPhen-2, with a score of 97.4% in the HumDiv database and 48.5% in HumVar (Figure 4) (15).

**Table 2:** Two different clinical manifestations of the same mutation—*GLA* c.493G>T (p.Asp165Tyr).

Characteristics	F20-A (16)	Case 1
Gender	Man	Man
Age at onset/diagnosis (year)	5/19	9/28
Clinical phenotypes	Classic	Classic
Family history	Positive	Positive
Angiokeratoma	No	Yes
Acroparesthesias	Yes	Yes
Hypohidrosis	Yes	Yes
Ocularchanges	Yes	Yes
Hearing loss	No	Yes
Proteinuria	Yes	Yes
Baseline eGFR	98.97	15.00
Left ventricular hypertrophy	No	Yes
Hypertension	No	Yes
Cerebrovascular	No	No
α-gal A (μmol/L/h)	0.19	0.37

F20-A refers to a previously reported patient from the Fabry Database (fabry-database.org) with the same mutation.

Lyso-Gb3 accumulation correlates with organ damage severity (5), while the impact of residual *GLA* activity on clinical phenotype remains debated (16, 17). In this study, both patients had identical *α*-GalA activity (0.37 µmol/L/h) but distinct clinical profiles (Table 3).Notably, Case 1's higher baseline Lyso-Gb3 (93.33 vs. 79.17 ng/mL) correlated with more rapid renal progression, underscoring Lyso-Gb3's role as a biomarker. Conversely, a previously reported patient (F20-A) with the same c.493G > T mutation showed milder manifestations despite lower *α*-GalA activity than Case 1 (Table 2).

**Table 3:** Comparison of clinical signs and symptoms between case 1 and case 2 at baseline and after approximately 3-4 years of agalsidase beta treatment.

Items	Case1 Baseline	Case1 Last follow-up	Case2 Baseline	Case2 Last follow-up
Sex	Male		Male	
Diagnosed age (years old)	28		36	
Last follow-up age (years old)	34		40	
*α*-GalA（*μ*mol/L/h）	0.37		0.37	
Plasma Lyso-Gb3（ng/mL）	93.33	27.38	79.17	24.93
Immunoglobulin G anti-agalsidase alfa antibody	Negative	Negative	Negative	Negative
Angiokeratoma	+（the whole body）	No significant change	+ (Left groin)	Partially disappeared
Small fiber neuropathy				
Sweating abnormality	Hypohidrosis	Improved	Hypohidrosis	Improved
Subjective Feeling	Elevated skin temperature	Frequency reduced	Normal	Normal
Pain	Distal limb pain	Frequency and degree reduced	Normal	Normal
Gastrointestinal symptoms	Left lower abdominal pain after eating; diarrhea	No pain; Frequency reduced	Diarrhea	Improved
Hearing symptoms	Tinnitus	Improved	Normal	Normal
Ocular symptoms	Occasional blurred vision	Normal	Normal	Normal
Cerebrovascular symptoms	Normal	Normal	P3-04 segment of right posterior cerebral artery (congenital)	
Cardiovascular events				
Cardiac symptoms	Slight interventricular septal thickening	Improved	LV posterior wall and septal thickening	Progress but slowly
Hypertension（mmHg）	149/93	Post-transplant: 115/65	103–125/49–59	105/65
Renal findings				
UREA（mmol/L）	14.32	Pre-transplant: unchanged; post: normal	Normal	Normal
CREA（μmol/L）	430	Pre-transplant: increased; post: normal	Normal	Normal
Urinary protein	++	Pre-transplant: ++; post: –	++	+

Biomarkers were measured with validated and consistent analytical methods across all follow-up visits.

Early ERT initiation is paramount before the onset of irreversible organ damage (6). The Fabry Outcome Survey advocates treatment before adolescence to prevent irreversible organ damage (18–20). Our patients started ERT at ages 30 (Case 1) and 37 (Case 2), after significant disease manifestation. Despite late initiation, both reported improvement in certain symptoms (e.g., neuropathic pain, gastrointestinal discomfort), suggesting that some clinical features may be partially reversible. However, our findings reinforce that ERT exerts limited efficacy in reversing advanced renal fibrosis and end-stage organ damage. This is best illustrated by Case 1's poor response during peritoneal dialysis, where persistent proteinuria and hypercreatininemia highlighted ERT's reduced efficacy in late-stage CKD. These cases reinforce that earlier ERT initiation—prior to the development of significant glomerulosclerosis—is associated with more favorable renal outcomes (4, 9, 21, 22).

A major concern is disentangling the contribution of each intervention from concurrent therapies. In Case 2, proteinuria improved while RAAS blocker doses remained strictly unchanged, indicating an add-on effect of ERT to stable RAAS blockade. In Case 1, dramatic renal recovery (creatinine normalization, proteinuria resolution) occurred only after kidney transplantation (performed after 21 months of ERT). Hence, transplantation was the primary driver of renal restoration, while ERT contributed to systemic substrate (Lyso-Gb3) reduction and graft protection, rather than reversing native renal failure. Notably, irregular ERT dosing in Case 2 due to social factors and economic pressure temporarily attenuated biomarker reduction and treatment efficacy, highlighting the critical importance of treatment adherence for optimal outcomes.

Beyond renal outcomes, both patients demonstrated systemic benefits of ERT. Case 2 showed partial regression of angiokeratoma, and both patients reported improvement in small fiber neuropathy-related symptoms (e.g., hypohidrosis, acroparesthesia, gastrointestinal discomfort). These findings align with studies demonstrating ERT-mediated Gb3 clearance in dermal and neural tissues (23, 24). However, Case 1's stable angiokeratomas may reflect advanced disease irreversibility, suggesting tissue-specific therapeutic thresholds. Ophthalmologic findings were satisfactory: Case 1 had no corneal deposits and stabilized retinal vasculopathy, which contrasts with a previous report showing no apparent protective effect of ERT on retinal vasculopathy (25).

The impact of ERT on left ventricular hypertrophy (LVH) remains contentious (26). Case 1 showed a reduction in interventricular septal thickness (12 mm → 11 mm) after 4.2 years of ERT and post-transplant blood pressure normalization, which is consistent with previous reports demonstrating that agalsidase beta effectively reduces myocardial hypertrophy in FD patients (27). In contrast, Case 2 exhibited slow progression of LVH (IVSd 12 mm → 13 mm) over 3.6 years of ERT, possibly due to shorter treatment duration or genetic modifiers. The absence of anti-drug antibody (ADA) monitoring is a notable limitation, as ADA may affect treatment efficacy and tolerability in individual patients (28, 29). Additionally, the lack of post-transplant renal biopsy precludes direct assessment of Gb3 deposition in the graft.

Despite these limitations, the safety profile of agalsidase beta was favorable in both patients. Both patients tolerated agalsidase beta without infusion-related adverse events, consistent with global safety profiles where severe reactions occur in <5% of cases (23, 30). This reinforces agalsidase beta's suitability for long-term use, particularly in adherent populations.

Beyond treatment-related considerations, this study also highlights important implications for family screening. The identification of affected family members in both cases (mother and daughter in Case 1; brother and daughter in Case 2) underscores the critical importance of cascade family screening following the diagnosis of a proband. This practice is essential for early diagnosis and timely institution of therapy in presymptomatic individuals, which can fundamentally alter the disease course (31, 32).

## Conclusion

This case series describes two Chinese adult male Fabry disease patients with proteinuria who received agalsidase beta for over three years. Both patients demonstrated stabilization of renal and cardiac function, improvement in systemic symptoms, and good tolerability. Proteinuria management required concomitant RAAS inhibition, particularly in patients with residual kidney function. Early initiation of agalsidase beta before irreversible organ injury remains key to optimizing prognosis. These cases also highlight the importance of family cascade screening for early diagnosis and intervention.

### Limitation

This study has several limitations. First, it is limited to two adult male patients with a retrospective design and no control group. Second, anti-drug antibodies (ADAs) were not measured, limiting evaluation of immunogenicity. Third, post-transplant renal biopsy was not performed to assess Gb3 deposition in the graft. Finally, the 3–4 year follow-up duration cannot rule out potential long-term progressive organ deterioration, particularly for cardiac and cerebrovascular outcomes. Multicenter prospective studies are warranted.

In summary, Case 1 displayed more typical and severe clinical features, including left lower abdominal pain, blurred vision, tinnitus, elevated skin temperature, and pain, symptoms that were absent in Case 2. Even when similar symptoms appeared in both, they were more pronounced in Case 1. Renal injury in Case 1 progressed to failure, and although ERT slowed this decline, it could not reverse the damage. Two years ago, the disease had advanced to CKD stage 5 with concurrent hypertension, necessitating kidney transplantation. In comparison, Case 2 was at CKD stage 2, and ERT resulted in improved proteinuria. Additionally, Case 1 had more extensive angiokeratomas and a higher frequency of diarrhea.

## Data Availability

The raw data supporting the conclusions of this article will be made available by the authors, without undue reservation.
